# Tributyltin and Zebrafish: Swimming in Dangerous Water

**DOI:** 10.3389/fendo.2018.00152

**Published:** 2018-04-10

**Authors:** Clemilson Berto-Júnior, Denise Pires de Carvalho, Paula Soares, Leandro Miranda-Alves

**Affiliations:** ^1^Grupo de Pesquisa, Desenvolvimento e Inovação em Endocrinologia Experimental-GPDIEEx, Instituto de Ciências Biomédicas, Universidade Federal do Rio de Janeiro, Rio de Janeiro, Brazil; ^2^Laboratório Integrado de Ciências Farmacêuticas (LICFAR), Universidade Federal do Rio de Janeiro, Rio de Janeiro, Brazil; ^3^Endocrinologia, Faculdade de Medicina, Universidade Federal do Rio de Janeiro, Rio de Janeiro, Brazil; ^4^Laboratório de Fisiologia Endócrina Doris Rosenthal, Instituto de Biofísica Carlos Chagas Filho, Universidade Federal do Rio de Janeiro, Rio de Janeiro, Brazil; ^5^Instituto de Investigação e Inovação em Saúde (i3S), Universidade do Porto, Porto, Portugal; ^6^Instituto de Patologia e Imunologia Molecular da Universidade do Porto (IPATIMUP) – Cancer Signaling and Metabolism, Porto, Portugal; ^7^Faculdade de Medicina, Universidade do Porto, Porto, Portugal; ^8^Departamento de Patologia, Faculdade de Medicina, Universidade do Porto, Porto, Portugal; ^9^Farmacologia e Química Medicinal, Instituto de Ciências Biomédicas, Universidade Federal do Rio de Janeiro, Rio de Janeiro, Brazil

**Keywords:** zebrafish, tributyltin, endocrine disruptors, imposex, obesogenic

## Abstract

Zebrafish has been established as a reliable biological model with important insertion in academy (morphologic, biochemical, and pathophysiological studies) and pharmaceutical industry (toxicology and drug development) due to its molecular complexity and similar systems biology that recapitulate those from other organisms. Considering the toxicological aspects, many efforts using zebrafish models are being done in order to elucidate the effects of endocrine disruptors, and some of them are focused on tributyltin (TBT) and its mechanism of action. TBT is an antifouling agent applied in ship’s hull that is constantly released into the water and absorbed by marine organisms, leading to bioaccumulation and biomagnification effects. Thus, several findings of malformations and changes in the normal biochemical and physiologic aspects of these marine animals have been related to TBT contamination. In the present review, we have compiled the most significant studies related to TBT effects in zebrafish, also taking into consideration the effects found in other study models.

## Introduction

Zebrafish, Danio rerio, is a native teleost to the southeastern Himalayan region that has emerged as a reliable model for studying not only embryogenesis and regeneration, but also disease. The main advantages of zebrafish when compared to other biological models refer to their small size, the easy maintenance characteristics, and relatively low cost (1). Zebrafish has a high fertility rate that is characterized by dozens of embryos per matching couple, which allow a significant number of genetic approaches, such as morpholino antisense oligonucleotide technology to knock down several genes, study their function, and generate new disease models (2). Zebrafish has also been used in the field of drug discovery with great success, since it can be used for target identification, pharmacokinetic/pharmacodynamic, and toxicology studies (3). Due to its large and traditional use in the drug discovery field, the expertise of zebrafish model has been transferred to the analysis of endocrine disruptor effects.

The anatomical structures are similar between zebrafish and human organs, which confirms that this model is versatile and useful. Compared to *Caenorhabditis elegans* and *Drosophila melanogaster* models, zebrafish has a greater number of genes with a higher homology to human genome (4). When it comes to *Mus musculus* comparison, zebrafish has about the same number of genes, although with less homology (70 versus 90%) but with a lower annual cost (4). Menke and coworkers showed the anatomic and histologic features of adult zebrafish, evidencing similarity in the hematopoietic system, spleen, thymus, heart, thyroid, kidney, gastrointestinal system, liver, pancreas, brain (with telencephalon, diencephalons, mesencephalon, metencephalon, and myelencephalon), hypothalamus, pineal gland, pituitary gland, eye, and musculoskeletal system tissues, besides reproductive organs (5).

Therefore, the use of zebrafish for toxicology investigation comprises reproductive, developmental, neuro, cardiac, ocular, endocrine, vascular, and carcinogenic toxicity with several end points to be analyzed that should be chosen carefully for each purpose (6). Thus, the use of zebrafish for studying the effects of endocrine disruptors and/or their mechanism of action is convenient.

Endocrine-disrupting chemicals (EDCs) are natural occurring or synthetic compounds that interfere with natural hormone synthesis, secretion, transport, binding, or elimination, leading to homeostatic imbalance (7). Gore et al. (2014) postulated that EDC can enter the human body by different routes of exposition, such as oral consumption of contaminated food or water, contact with skin and/or inhalation, intravenous administration, and biological transfer through the placenta or milk during lactation (8).

As one of the most widespread EDC, tributyltin (TBT) has gained special attention. TBT is an organotin (one or more covalent bonds between carbon and tin atoms) that is used as an antifouling agent in boat paints and is continuously released into the water. As a result, harbor areas are deeply affected by this compound, which causes changes in the endocrine system of marine organisms, such as the development of male sexual anatomical characteristics in female gastropods, leading to sterility and death (9). TBT is rapidly absorbed by marine organisms, incorporated and accumulated in different tissues; after absorption, TBT can be metabolized and can generate other tin molecules, with different toxic properties and mechanisms of action (10).

The studies regarding TBT effects in zebrafish are rare compared to other species and EDC. Li and coworkers showed that the exposure of common carp to TBT for 7 days leads to oxidative stress, the inhibition of antioxidant enzymes, and the inhibition of the Na^+^/K^+^ ATPase activity, acetylcholinesterase, and monoamine oxidase (11). Also, a diminished activity of Na^+^/K^+^ ATPase was found in *Sebastiscus marmoratus*, which corroborates with the idea of a toxic effect of TBT (12).

### TBT, Gonads, and Sexual Bias

**Figure 1:**
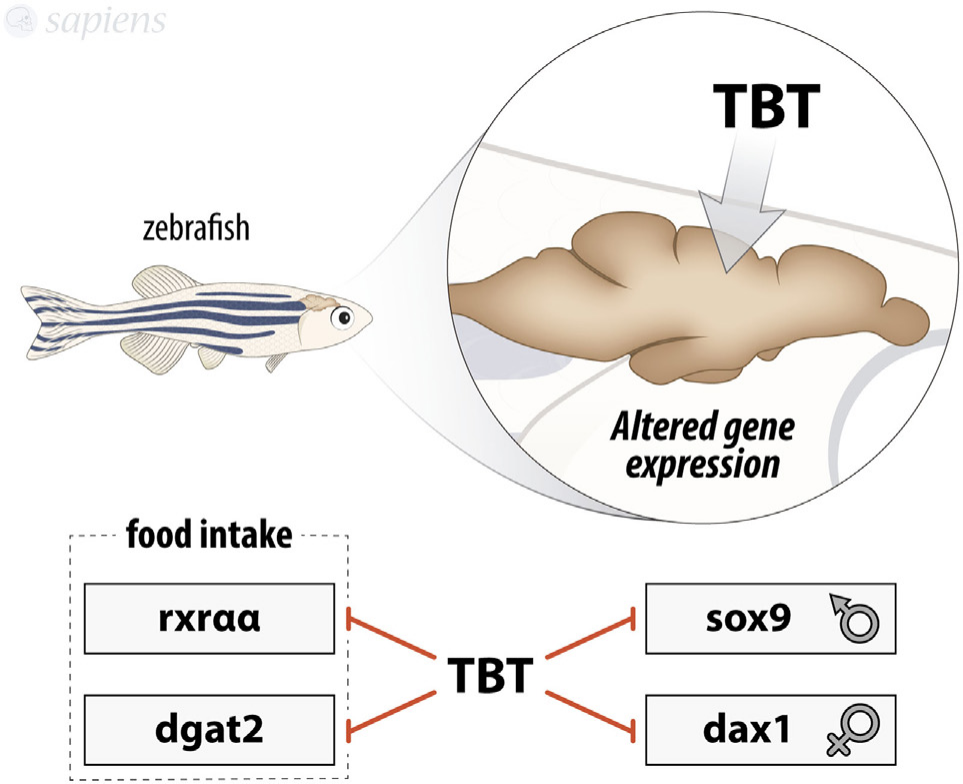
Summary of tributyltin (TBT) effects on zebrafish brain. TBT is able to modulate gene expression in zebrafish brain by decreasing rxraa, dgat2, sox9, and dax1 mRNA expression.

Regarding sexual development, intraperitoneal injections of 1 or 5 mg/kg TBT in adult zebrafish lead to the reduction in mRNA levels of *sox9* and *Dax1* in brain, which is a conflicting result (13). TBT as a male-biased population agent usually causes a severe shift in organism end point toward masculinizing phenotype (14). *sox9* gene encodes a transcription factor related to the male phenotype, while *Dax1* encodes a nuclear receptor that acts in the female development (15), so the presence of lower levels of *sox9* in the brain, together with a male phenotype animal, shows how complex EDC treatment effects could be (Figure 1).

**Figure 2:**
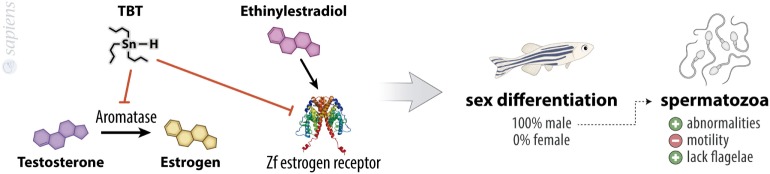
Tribultytin (TBT) acting in sexual bias. TBT is an inhibitor of aromatase, the enzyme responsible for testosterone to estrogen conversion, besides inhibiting zebrafish estrogen receptor, decreasing the effects of ethinylestradiol. These molecular events point to male sexual differentiation of almost 100% of treated animals. TBT-treated animals also present increased spermatozoa abnormalities, increased lacking flagellae spermatozoa, and a decreased spermatozoa motility.

Tributyltin promotes a dose-dependent increase in the masculinization rate of embryos treated for 70 days from hatching, reaching almost 100% of sex rate toward male with the concentration of 100 ng/L. These animals show abnormalities and a decreased motility of spermatozoid, because this population produces a higher quantity of spermatozoids that lack flagella (16). This is in agreement with other reports in the literature which suggest that TBT is an imposex-inducing agent in other species (17–23) and with the finding of aromatase inhibitory ability of TBT. Aromatase is the enzyme responsible for the conversion of androgens into estrogens in cells (Figure 2). Considering this, the human granulosa-like tumor cell line KGN displayed a significant suppressed aromatase activity when treated with TBT (24). Also, TBT might function as an agonist of the estrogen receptor alpha (ERα), since it has a proliferative effect on ER (+) breast adenocarcinoma cell line (MCF-7) (25). The treatment of HeLa cells transiently co-transfected with zebrafish estrogen receptors (zfERα, zfERβ1, and zfERβ2) with ethinyl estradiol results in a fourfold to sixfold increase in luciferase activity, an effect that was inhibited by TBT. By contrast, when cells were co-transfected with zebrafish androgen receptor and treated with testosterone, the treatment with TBT was not able to change luciferase activity, showing that imposex-inducing ability of TBT is widely complex and a multistep action (13).

### The Obesogenic Role of TBT

Besides imposex, TBT is highly associated to increased adipogenesis and is considered as obesogenic (26). Little is known about TBT effects in brain, most of the studies being focused on gene expression alterations concerning enzymes involved in lipid metabolism and sexual hormones (13, 27). Studies using 10 or 50 ng/L of TBT for 9 months in male and female animals showed the modulation of *R*etinoid *X R*eceptor alpha (RXRα/α)-nuclear receptor and *D*iacyl*G*lycerol O-*A*cyl*T*ransferase 2 (DGAT2)-lipogenic enzyme in both genders, with no modulation of PPARγ levels in brain, besides gender-specific alterations of gene expression (Figure 1). TBT might exert its lipogenic and adipocyte differentiation effects through the well-known RXR–PPARγ complex ligand ability (28, 29). These results confirm zebrafish as a good model for studying lipid homeostasis, since the complex mechanisms underlying food intake control and obesity development are similar to mammals.

The role of TBT as an obesogenic factor is well documented in the literature. Li and coworkers showed an activation of RXR-PPARγ heterodimer, triglyceride storage, and expression of adipogenic marker genes even in the presence of PPARγ agonist GW9662 in cultured preadipocytes (30). Indeed, TBT was shown to bind not only to RXR but also to PPARγ receptor (31), leading to weight gain, altered lipid homeostasis, lipid accumulation, raised expression of the adipocyte marker C/EBPα, reduced adiponectin expression, altered glucose metabolism, increased PPARγ expression, and hepatic inflammation (32–34).

Zebrafish treated with TBT shows an increase in adipogenesis at 15 days post fertilization and displays significantly increased adipocyte differentiation markers, with altered gene expression profile of adipogenic factors, like POMC (hypothalamic factor involved in feed behavior) and leptin (35). These data are consistent with the findings showing that female rats treated with TBT for 15 days present hyperleptinemia (36).

**Figure 3:**
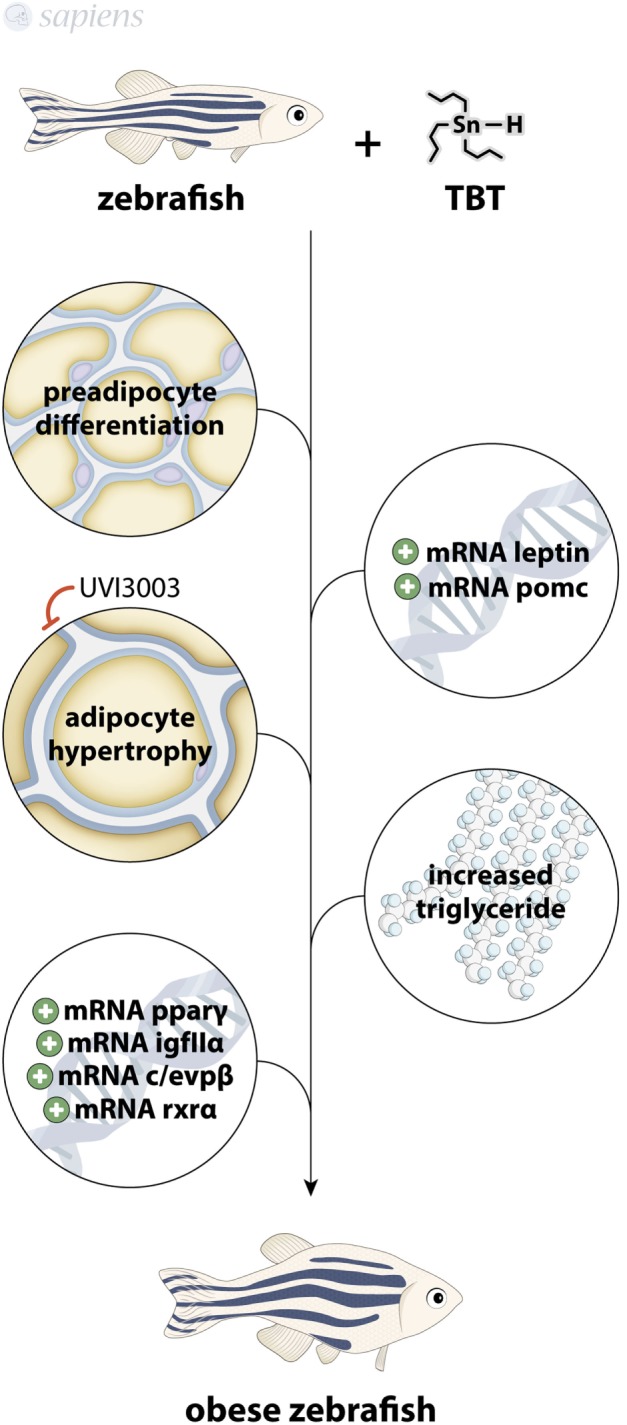
Tributyltin (TBT) as obesogenic molecule. TBT-treated zebrafish presents increased preadipocytes differentiation, modulation of pomc, leptin, pparγ, c/evpβ, IGFIIα, and rxrα mRNA, increased adipocyte hypertrophy (that can be blocked by UVI3003) and increased triglyceride levels, culminating in an obese animal.

Exposure to TBT in the nanomolar range for 3 days increases the percentage of adiposity in larvae (by Nile red staining of adipocyte lipid droplets) with the induction of adipocyte hypertrophy despite fasting (37). Interestingly, human PPARγ antagonists did not block the *in vivo* obesogenic effect of TBT, but the human RXR antagonist UVI3003 fully abolished the effect, confirming that zebrafish adipose tissue is readily responsive to adipogenic molecules, even in a fasting state *via* RXR pathway (38). Zebrafish exposed to TBT for 9 months also presented altered body weight with increased triglycerides in male and the modulation of a range of lipogenic genes in liver, such as PPARγ, RXRα, C/EBPβ, and IGFIIα, all of them being adipogenic stimulators (27). Some recent work fully ratifies not only the zebrafish as an animal model for adipose tissue studies but also points to new techniques for assaying adipocytes dynamics in zebrafish (39–41) (Figure 3).

It was also reported that TBT could affect nutritional status by modifying yolk absorption. Yolk provides energy and nutrients for developmental phases in teleosts, since it is mainly composed of phospholipids and triacylglycerols packed into lipoprotein particles (vitellogenin) and surrounded by the yolk syncytial layer that functions to hydrolyze yolk molecules and transport them to embryos. TBT, as an obesogenic agent, causes a faster uptake of yolk (42).

### Other TBT Effects in Zebrafish

Regarding behavioral aspects, there are only few studies and most of them point to altered end points. Male Wistar rats treated with various doses of TBT showed a dose-dependent decrease in spontaneous motor activity during dark phase and an inhibition in the acquisition of shock avoidance responses also in a dose-dependent manner, indicating that TBT exposure can cause a significant disturbance in rat behavior (43). Non-reproductive behavior alteration in teleost rare minnow was also documented, revealing that fish exposed to TBT had less group cohesion during the course of a 10min period of observation, altered shoaling in novel tank test, shorter latency before leaving shoal mates, and they spent more time away from shoal than control fish, with increased anxiety (44).

Considering the antioxidant ability and immunity, an 8-week treatment with TBT reduced superoxide dismutase (SOD), catalase (CAT), and glutathione peroxidase (GPX) activities in a dose-dependent manner, with an increase in the relative expression of HSP70 and HSP90, IL-1β, IL-6, TNF-α, and NF-κB. Thus, TBT is an inducer of oxidative stress and plays an important role in the positive modulation of pro-inflammatory cytokines (45). This is consistent with data showing a decreased activity of SOD, CAT, and GPX in other species (46, 47), a higher expression of HSP70 in common carp (48), an increased IL-1β secretion by human immune cells (49), an increased IL-6 production in human peripheral blood mononuclear cells (50), and higher TNF-α levels in mouse serum (51).

It was also reported that TBT could affect nutritional status by modifying yolk absorption. Yolk in teleosts provides energy and nutrients for the developmental phase, being composed in majority of phospholipids and triacylglycerols packed into lipoprotein particles (vitellogenin) and surrounded by the yolk syncytial layer that functions hydrolyzing yolk molecules and transporting them to embryo. TBT, as obesogenic agent, caused a faster uptake of yolk in an automatic method to segment and quantify yolk areas in zebrafish larvae (42).

Zebrafish larvae treated with TBT (0.03 nM) show increased death with diminished hatch rates, an abnormal body curvature, a higher pericardial edema, and a dorsal curve rate. These data are controversial since Liang and coworkers (52) showed a higher hatch rate in embryos treated with higher concentrations of TBT (1 nM). Nevertheless, this could be due to EDC dose–response behavior that often show nonmonotonic dose–response curve in a U-shaped or inverted U-shaped curves (0.03 or 1 nM), probably belonging to any point of the curve with a hatch rate as end point (53). Also, a decrease in heart rate was reported, with the differential expression of important genes related to cardiac function and development, such as *cav3* that encodes caveolin 3 protein and *cmlc1*, which encodes cardiac myosin light chain-1 (essential for zebrafish cardiogenesis) (54, 55). Other studies concerning cardiac function in TBT-treated animals were published revealing that this organotin induces cardiomyopathy in clam Ruditapes (56) and increased collagen deposition in heart interstice, impaired coronary vascular reactivity to estradiol, and enhanced the number of mast cells proximate to cardiac vessels in rats (57).

Unprecedented studies in zebrafish assessing TBT effects in systems not widely rummaged are also available. TUNEL staining of zebrafish embryos displayed TBT-induced apoptosis restricted to retinal neuronal cells and unidentified cells around trigeminal neurons with macrophage accumulation, probably by higher accumulation of TBT in the optic tract (58), showing selective apoptosis in this tissue (59). Also, genotoxicity using zebrafish erythrocytes was reported in an erythrocytic nuclear abnormality (ENA) frequency assay in animals exposed for 4 months, exhibiting a higher ENA frequency in TBT-treated conditions (60).

## Conclusion

Studies concerning TBT as an EDC are rapidly growing every year based on its wide range of effects in humans and laboratory animals. These broad options of models comprising normal systems and diseases are of great importance for recognizing TBT actions due to its widespread usage in the world. Zebrafish is a reliable model for studying several diseases like cancer, obesity, and inflammation and has become a robust tool for assessing EDC effects. Studies using zebrafish as a biological model to access TBT effects are few but they corroborate the effects found in other classical animal models, such as murine ones. Brain effects of TBT related to behavior changes are well documented in the literature (44, 61–64) and absent in zebrafish, even though these animals possess similar structures and molecular complexity comparable to other models in order to test memory, anxiety, fear, and social behavior (65–67). Also, considering the hypothalamus–pituitary–thyroid axis, no study has been done yet to evaluate the effects of this compound in zebrafish, although an extensive and elucidating review described the action of TBT in other species (68).

## Author Contributions

CB-J, DC, PS, and LM-A conceived and wrote the article.

## Conflict of Interest Statement

The authors declare that the research was conducted in the absence of any commercial or financial relationships that could be construed as a potential conflict of interest.
